# 6-Prenylnaringenin—Its Beneficial Biological Effects and Possible Applications

**DOI:** 10.3390/ijms262110662

**Published:** 2025-11-01

**Authors:** Tomasz Tronina, Daniel Łój, Jarosław Popłoński, Agnieszka Bartmańska

**Affiliations:** Department of Food Chemistry and Biocatalysis, Wrocław University of Environmental and Life Sciences, Norwida 25, 50-375 Wrocław, Poland; daniel.loj@upwr.edu.pl (D.Ł.); jaroslaw.poplonski@upwr.edu.pl (J.P.)

**Keywords:** 6-prenylnaringenin, hop prenylflavonoid, health benefits, GABA_A_ positive allosteric modulator, antimicrobial, cancer prevention, phytoestrogen

## Abstract

6-Prenylnaringenin (6-PN) is a natural compound which occurs in some plants, but the primary dietary source for humans is beer. This compound exhibits broad and potent antimicrobial, anticancer, and neuroactive properties, and weak estrogenic effects. Currently, hop extracts standardized for 6-PN content (relative to other prenylflavonoids) are commercially available and utilized in the non-hormonal treatment of menopause. It is probable that in the future, 6-PN will be employed in the prevention or treatment of non-hormone-dependent cancers and infectious diseases, as well as a sedative, hypnotic, and analgesic agent. Further research is essential to precisely determine the exact mechanisms of action of 6-PN and, critically, to leverage its unique therapeutic profile. This review synthesizes current evidence, highlighting that 6-PN warrants priority investigation as a core scaffold for novel drug development, particularly as a GABA_A_ positive allosteric modulator and a synergistic antimicrobial agent, potentially offering a safer alternative to more potent phytoestrogens found in hops.

## 1. Introduction

C-prenylated flavonoids are a group of compounds where the flavonoid backbone is linked to a lipophilic prenyl side chain. This prenylation typically occurs at the C-6 and/or C-8 positions in the A ring, or at the C-3′ and/or C-5′ positions in the B ring [1].

Prenylation is known to enhance the antibacterial, anti-inflammatory, antioxidant, cytotoxic, insecticidal, and estrogenic activities of flavonoids [2]. The prenyl side chain can increase the binding affinity of flavonoids to P-glycoprotein, significantly improving their biological activity [3], including their anticancer properties [4]. Furthermore, prenylated flavonoids often act selectively, exhibiting a higher cytotoxic effect against cancer cells than against normal cells. However, the presence of a non-polar prenyl group in the flavonoid molecule has the adverse effect of reducing their water solubility, which in turn negatively impacts their bioavailability and reduces their absorption [2]. Consequently, this limits the therapeutic potential of these valuable biologically active compounds.

**Figure 1:**
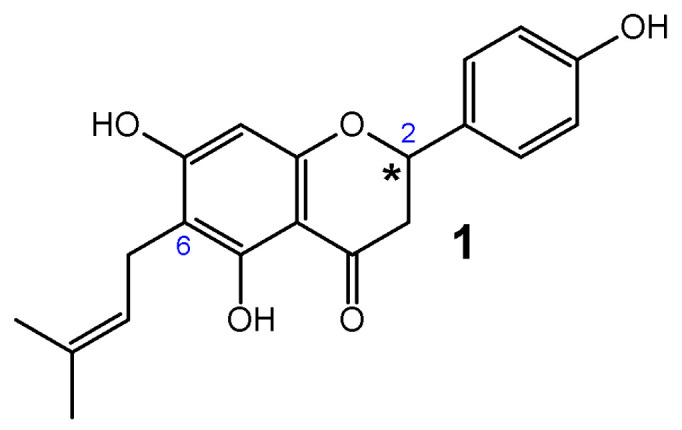
Structure of 6-Prenylnaringenin (6-PN) (**1**). An asterisk indicates the chiral center in the compounds.

6-Prenylnaringenin (6-PN) (**1**) (2,3-dihydro-5,7-dihydroxy-2-(4-hydroxyphenyl)-6-(3-methyl-2-butenyl)-4H-1-benzopyran-4-one) is classified as a prenylated flavanone with three hydroxyl groups located at C-5, C-7, and C-4′, and the prenyl group at C-6 (Figure 1).

In its pure form, compound **1** presents as a crystalline pale yellow solid with a melting point of 209–209.5 °C. Due to the additional prenyl group, it exhibits poor solubility in water (1.55 mg/L) [5] but is readily soluble in solvents such as chloroform, dichloromethane, ethyl acetate, DMSO, and acetone [6].

Dhooghe et al. developed an accessible and highly accurate HPLC-DAD method for determining prenylflavonoids in hop extract and capsules, which employed quercetin and naringenin as secondary standards. The use of secondary standards is a valuable solution when quantifying components that are either commercially unavailable or prohibitively expensive [7].

6-PN (**1**) is a chiral compound (2*R*, 2*S*) and can be quantified using enantiospecific LC-ESI-MS on a Chiralpak^®^ AD-RH column using isocratic elution. Quantitative MS data were obtained by monitoring selected [M-H] ions for both enantiomers of 6-PN (**1**) and the internal standard, 4-acetamidobenzoic acid. This approach allows for the precise enantiospecific quantification of compound **1 [8]**.

### 1.1. Occurrence

6-PN (**1**) was initially described in *Sophora tomentosa* L. [9]. It has also been identified in various other plants, including hops (*Humulus lupulus* L.) [10], *Wyethia glabra* [11], *W. invenusta* [12], *Lupinus luteus* [13], *Glycyrrhiza glabra* [14], and *Psoralea corylifolia* [15]. The primary natural source of 6-PN (**1**) in the human diet is hops. Hop cones contain a small amount of 6-PN (**1**) (approximately 0.004% of dry weight) [16], while hop extracts used in brewing are much richer in 6-PN (**1**) (Table 1).

Hops traditionally used in beer production have been valued as a medicinal herb since ancient Egyptian times [21,22]. The earliest recorded medicinal use of hops dates back to an 11th-century book, where the Arab physician Mesue described the anti-inflammatory properties of this perennial herb [23].

**Figure 2:**
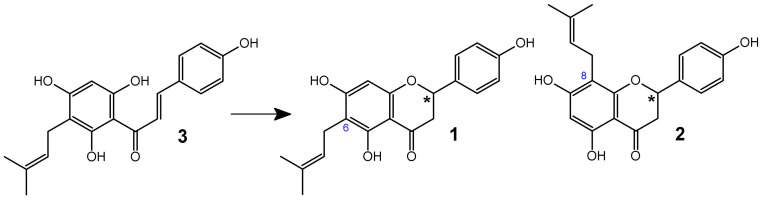
Transformations of desmethylxanthohumol (**3**) to 6-PN (**1**) and 8-PN (**2**) during the beer production process. Asterisks indicate the chiral center in the compounds.

Subsequently, the German Commission E, which is a scientific advisory board of the Federal Institute for Drugs and Medical Devices, also approved hops for addressing “mood disorders such as anxiety and restlessness, and sleep disorders” [24,25]. Currently, *Humulus lupulus* L. (hops) is recognized as a source of many valuable compounds with notable antibacterial activity [26,27,28] and significant pharmaceutical potential [29,30]. The main source of 6-PN (**1**) in the human diet is beer. However, the presence of 6-PN (**1**) in beer is not solely due to its content in hops, but also as a result of the isomerization of desmethylxanthohumol (**3**) to 6-PN (**1**) during the hop wort boiling process. Desmethylxanthohumol (DXN) (**3**), a prenylated chalcone found in hops, is transformed into a mixture of phytoestrogens: 6-prenylnaringenin (**1**) and 8-prenylnaringenin (8-PN) (**2**). This conversion is facilitated by the presence of two free hydroxyl groups at the C-2′ and C-6′ positions of desmethylxanthohumol. While 6-PN (**1**) and 8-PN (**2**) are formed in small quantities during the drying, storage, and extraction of hops, the conversion process from DXN (**3**) to 6-PN (**1**) and 8-PN (**2**) is particularly rapid when the wort is boiled in a brewhouse [16,31] (Figure 2).

The 6-PN (**1**) content in beer varies significantly, influenced by both the brewing process and the specific beer brand [32,33]. The concentration of 6-PN (**1**) can differ widely, also depending on the analytical method employed. For instance, research by Stevens and Page demonstrated a range from 1 µg/L in European lager to 240 µg/L in American porter [32]. While the aforementioned concentrations of 6-PN (**1**) might appear to have marginal effects (Table 2), they are nonetheless believed to offer beneficial health effects, a point that warrants further investigation. Simultaneously, it is important to acknowledge the known harmful side effects associated with alcohol consumption in beer [34,35,36]. Moreover, per capita beer consumption varies significantly by country, for instance, with the highest levels observed in the Czech Republic—152.1 L in 2023 [37].

### 1.2. Pharmacokinetics of 6-PN (***1***)

The metabolism and mode of action of all prenylated flavonoids are not yet fully understood, particularly regarding the biotransformation of these compounds, which are present in small quantities in hops. Only a few studies have assessed the absorption, metabolism, and excretion of 6-prenylnaringenin (**1**) or its structural isomer 8-prenylnaringenin (**2**), both derived from hop extracts, in humans. There are individual differences in the biotransformation of compound **1**, and its absorption is notably weaker than that of 8-PN (**2**) [39,40,41,42,43].

Van Breemen et al. observed that 6-PN (**1**) and 8-PN (**2**) exhibit low oral bioavailability and significant inter-individual variability. Following a single oral dose of 500 mg of either compound, both were rapidly absorbed and extensively metabolized in both sexes. Notably, 6-PN (**1**) was found to be four to five times less bioavailable than its isomer, 8-PN (**2**). Despite this lower bioavailability, with mean C_max_ values for 6-PN (**1**) ranging from 483 to 602 nmol/L compared to 2250–3418 nmol/L for 8-PN (**2**), 6-PN (**1**) demonstrated similar bioactivity to 8-PN (**2**). Pharmacokinetic and biotransformation studies of pure 6-PN (**1**) revealed its rapid conjugation with glucuronic acid. Furthermore, both prenylflavonoids exhibited promising ex vivo immunostimulatory effects [39]. Calvo-Castro et al. also investigated the bioavailability and safety of orally administered 6-PN (**1**) and 8-PN (**2**) in sixteen healthy young subjects, as well as their effects on peripheral blood mononuclear cells (PBMC). Their experiments confirmed previous findings, demonstrating that both compounds were equally effective in increasing PBMC viability [44].

The significant differences observed in the absorption, metabolism, elimination, and biological activity between these two structurally very similar positional isomers warrant further comprehensive study.

## 2. Biological Activity

Prenylflavonoids are a fascinating class of naturally occurring substances known for their diverse and desired biological properties [45,46,47,48,49]. While prenylation often enhances certain bioactivities of flavonoids, particularly their estrogenic and anticancer effects, it also tends to reduce their bioavailability and increase their bioaccumulation in tissues [49,50]. Despite these challenges, their promising biological activity and favorable safety profiles suggest that prenylated flavonoids hold significant potential for use as nutraceuticals or drugs [51,52,53].

While numerous reviews exist for prenylflavonoids generally, research on 6-PN (**1**) often lacks a unified focus due to its presence alongside the highly potent phytoestrogen 8-PN (**2**). This review aims to critically evaluate the current literature on 6-PN’s non-hormonal properties—including its potent neuroactive, antimicrobial activities as well as other potentially beneficial health effects—to demonstrate its potential as a safer therapeutic agent compared to both conventional hormone replacement therapy (HRT) and more potent phytoestrogens.

### 2.1. Antioxidant Activity

The antioxidant effect of flavonoids stems, in part, from the presence of -OH functional groups in the molecule, which can scavenge free radicals and chelate transition metal ions. This property makes these compounds a subject of significant research regarding their potential anticancer effects [54,55,56]. 6-PN (**1**), like other prenylflavonoids, positively impacts human health, largely attributed to its antioxidant properties. However, relatively few studies on this specific aspect have been published, likely due to its low dietary content [57]. The conducted research indicates that most analyzed compounds exhibit high levels of antioxidant activity, though inhibitory effects and structure-dependence have also been observed [2,17,47]. Direct comparisons between compounds are challenging due to the variety of measurement methods and positive controls used by different research groups. Implementing validation in future studies could yield more reliable information. For instance, studies on antioxidant activity have shown that 8-PN (**2**) was identified as an antioxidant, while 6-PN (**1**) appeared to be a mild pro-oxidant in certain assays, such as the DMPD chemiluminescence assay (based on DMPD^•+^ scavenging activity) and the LDL oxidation inhibition assay [58,59]. Therefore, the precise mechanism of the antioxidant activity of these compounds still requires further elucidation.

### 2.2. Phytoestrogen Activity

Estrogens have been shown to offer several beneficial effects on human health, including protection against menopausal symptoms, osteoporosis, cardiovascular disease, and some potentially neurodegenerative disorders. However, estrogens are also identified as one of the most significant risk factors for breast cancer [60]. Phytoestrogens are non-steroidal compounds derived from plants that elicit a biological response similar to that produced by the primary human estrogen, 17*β*-estradiol, in hormonal assays. Flavonoids represent the main group of phytoestrogens associated with this physiological effect [61].

During hop research, it was found that desmethylxanthohumol (**3**) is a pro-estrogenic substance [62]. Hänsel and Schulz were the first to accurately describe the chemical structure of desmethylxanthohumol (**3**) derivatives. However, they concluded that while the mixture of 8-prenylnaringenin (**2**) and 6-prenylnaringenin (**1**) exhibited an estrogenic effect, 6-PN (**1**) alone did not [63]. Milligan et al. isolated and characterized 8-PN (**2**) as the major estrogenic substance in hops, considering it one of the most potent plant estrogens known [64].

In vitro studies have shown that 8-PN (**2**) mimics the action of 17*β*-estradiol, although its potency as an estrogen is considerably lower (10–20,000-fold) [65,66,67,68,69]. The estrogenicity of other structurally related hop flavonoids was less than 1% compared to 8-PN (**2**), decreasing in the following order: 8-prenylnaringenin (**2**) >> 6-prenylnaringenin (**1**) > 8-geranylnaringenin > 6,8-diprenylnaringenin [65].

Menopause is defined as the permanent cessation of menstruation, directly resulting from the termination of ovarian follicle activity [70]. The standard treatment for menopause involves hormone replacement therapy (HRT), the use of selective estrogen receptor modulators [71], and other medications, such as selective serotonin reuptake inhibitors, which help improve vasomotor symptoms [72].

The use of botanical dietary supplements as an alternative to hormone replacement therapy (HRT) has recently increased [73,74]. This trend is partly driven by findings from the Women’s Health Initiative (WHI), which showed a relationship between long-term HRT use and an increased risk of developing hormone-dependent cancers [75,76].

Effenberger et al. demonstrated that 6-PN (**1**) might serve as an alternative to conventional hormone replacement therapy for preventing osteoporosis due to its preference for ER*β* (estrogen receptor beta). However, its safety is dose-dependent, with cytotoxic effects observed at high concentrations (≥10^−4^ M) [77]. The clinical utility of 6-PN (**1**) in managing menopausal symptoms and osteopenia is primarily derived from its preferential activity toward ERβ. Crucially, its significantly weaker estrogenic effect compared to 8-PN (**2**) [65] makes it a particularly attractive and safer candidate for alternative hormone therapy, reducing the potential risks associated with stimulating estrogen-receptor alpha (ERα)-positive tissue proliferation—a known concern with potent phytoestrogens in susceptible individuals.

*Humulus lupulus* L. (hops) is a popular plant source for dietary supplements, particularly used by women to alleviate postmenopausal symptoms. Despite its popularity, the benefit–risk ratio and the precise composition of hop extract remain an area of ongoing research (Table 3).

Clinical trials are crucial for evaluating the effectiveness of 6-PN (**1**) in alleviating menopause symptoms and ensuring its safe use. A comprehensive understanding of 6-PN’s (**1**) impact on the human body will enable its most beneficial application in treating postmenopausal symptoms while minimizing side effects. This knowledge will also facilitate the optimal selection of dosage and therapy duration.

### 2.3. Anticancer Activity

Cancer stands as the second leading cause of death globally. In 2022, an estimated 20 million new cancer cases and 9.7 million deaths were reported [78]. Among all cancers, the most frequently diagnosed types include lung (12.7%), breast (10.9%), colorectal (9.7%), and gastric cancer (7.81%). Cancer cells are distinct from normal cells due to their uncontrolled proliferation, dedifferentiation, and loss of function, invasiveness, and metastasis [79,80].

Although numerous therapies currently exist, significant emphasis is placed on naturally derived compounds. This is due to their often high bioavailability, cost-effectiveness, and minimal side effects. Flavonoids, for instance, demonstrate anticancer effects by regulating various molecular targets. These include cell cycle blocking, DNA repair, anti-inflammatory effects, activation of tumor suppressor genes and oncogene suppression, regulation of hormone levels and growth factors, inhibition of invasion, proliferation, angiogenesis, and metastasis, and induction of apoptosis [81].

Some prenylflavonoids can directly inhibit the growth of cancer cells while exhibiting low toxicity towards healthy tissues. Due to their antioxidant effects, anti-inflammatory properties, and ability to modulate the metabolism of carcinogenic substances, some of these compounds may even be utilized in cancer prevention [82,83,84]. Interestingly, certain compounds can affect cancer cells even when classical chemotherapy fails. Among these is 6-PN (**1**), which acts as both a substrate and inhibitor of the efflux transporter breast cancer resistance protein (BCRP/ABCG2). BCRP/ABCG2 is a membrane-bound multidrug transporter often overexpressed in cancer cells, contributing to their resistance to conventional treatments [85]. Although not as active as chalcone xanthohumol (**4**), a major prenylflavonoid found in hops, 6-PN (**1**) demonstrates anticancer activity through multiple pathways. Its anti-cancer properties are summarized in Table 4.

Currently, the impact of prenylnaringenin 6-PN (**1**) on the risk of developing estrogen-dependent cancers remains insufficiently explained and documented. Phytoestrogens are thought to be linked to the etiology of breast cancer and are being evaluated as either potential chemopreventive agents or cancer promoters [95]. For example, the highly estrogenic 8-PN (**2**) has been shown to increase uterine wet weight in rat experiments, which suggests that it may promote hormone-dependent tumors [96,97].

Phytoestrogens may act as chemopreventive agents, while also potentially promoting the growth of cancer cells via activation of the estrogen receptor. Furthermore, they can exert estrogenic effects via both receptor-dependent and receptor-independent mechanisms. Activation of ERα is associated with proliferative responses in the mammary gland and uterus. These findings suggest that phytoestrogen intake might not be suitable for patients at an increased risk of hormone-dependent cancers or for cancer survivors [98]. Molecular studies have demonstrated that almost all popular herbal supplements contain phytochemicals capable of binding to the human estrogen receptor. Phytoestrogens could be effective growth promoters for estrogen receptor-positive tumors and may also pose a risk to patients with ER-positive tumors who are undergoing antiestrogen treatment [99].

In contrast to 8-PN (**2**), neither 6-PN (**1**) nor hop extract produced such a tumor-promoting effect. Wang et al. explained that 6-PN (**1**), acting as an aryl hydrocarbon receptor (AHR) agonist, and hop extract enhance the non-toxic estrogen 2-hydroxylation pathway by increasing P450 1A1 expression via AHR in MCF-10A and MCF-7 cell lines [94]. 6-PN (**1**) increased the expression of the tumor suppressor gene (AHRR) and genes involved in estrogen metabolism (CYP1A1, CYP1B1). Although 6-PN (**1**) can activate both the detoxification and genotoxic pathways of estrogen metabolism, the hop extract as a whole only modulates the genotoxic pathway by increasing CYP1B1 mRNA expression.

These data highlight the significant role of 6-PN (**1**), found in hop extract, as a potential modulator of estrogen metabolism. This is attributed to its agonist effects on both ERα and AHR [100]. Hitzman et al. further confirmed that 6-PN (**1**) from hops interferes with the regulation of CYP1A1 via ERα, thereby facilitating estrogen detoxification [101]. While hop extracts primarily modulate the genotoxic pathway of estrogen metabolism, they also contain phytoestrogens like 8-PN (**2**). Since hop supplements are frequently used by women to alleviate postmenopausal symptoms, caution and further research are warranted regarding the use of hop preparations. This is particularly important due to the role of estrogenic compounds in the development of estrogen-dependent cancers, including endometrial cancer.

The results gathered thus far highlight the significant role of 6-PN (**1**), present in hop extract, as a potential modulator of estrogen metabolism. This suggests a potentially protective role in reducing the risk of breast cancer and underscores the importance of standardizing botanical extracts for safe use. Therefore, hop extracts intended for use should contain precisely defined and adjusted doses of desired bioactive phytochemicals, including the chemopreventive xanthohumol (**4**). Crucially, they must not contain phytochemicals that could expose individuals to adverse side effects. However, for medicinal purposes, using pure compounds appears to be a safer approach.

The identification of 6-PN’s (**1**) broad potential against both estrogenic and non-estrogenic cancer cells offers promising clinical prospects for its use to enhance the effects of pharmacological drugs. However, caution is advised when using even weak phytoestrogens in individuals at risk of hormone-dependent cancers.

Chemical modifications to compounds based on the initial 6-PN (**1**) structure could potentially lead to enhanced pharmacological effects.

In summary, the presented data suggest that 6-PN (**1**) is a promising candidate for use as an active substance in chemoprevention. It could also serve as a valuable model structure for developing novel epigenetic prevention and therapy strategies for various cancers, including melanoma.

### 2.4. Antimicrobial Activity

Antimicrobial properties of flavonoids strictly depend on the compound’s structure [102,103]. Literature data indicate that the saturation of the double bond at the C2–C3 position, the presence and position of hydroxyl groups (at C-5, C-7, and C-4′) [104,105,106], as well as the presence of additional substituents such as prenyl groups (primarily at the C6 position, and also at C8) [107,108] are crucial for the antibacterial properties of flavonoids.

#### 2.4.1. Antibacterial Activity

Multidrug-resistant microorganisms are a leading cause of infectious diseases globally [109]. The most recent global estimates on antibiotic resistance highlight three human pathogens as particularly concerning worldwide due to their association with nosocomial and community-acquired infections: *Staphylococcus aureus*, *Escherichia coli*, and *Klebsiella pneumoniae* [110]. Approximately 50% of patients hospitalized with diabetic foot infections suffer from osteomyelitis, which is often linked to MRSA (Methicillin-resistant *Staphylococcus aureus*) [111]. The poor diffusion of antibiotics into necrotic tissues, primarily due to persistent biofilm formation, poses significant challenges in clinical practice [112]. A promising strategy for addressing the problem of antibiotic resistance in certain bacterial strains is to exploit the synergy between natural compounds, including flavonoids and traditional antibiotics [113]. Hop phenolic compounds, with their dual antibacterial and anti-biofilm effects, may offer a new perspective in treating MRSA-caused infections [114].

The antimicrobial activity of 6-prenylnaringenin (**1**) was first demonstrated by Mizobuchi and Sato [10]. 6-PN (**1**) inhibited the growth of dermatophytes *Trichophyton mentagrophytes* and *T. rubrum* more effectively than the positive control griseofulvin (MIC: 3.13 µg/mL) [10] (Table 5).

Shirataki et al. studied the activity of 6-PN (**1**) against Gram-positive bacteria of the genera *Bacillus* and *Staphylococcus* as well as Gram-negative bacteria of the genera *Escherichia*, *Helicobacter*, *Klebsiella*, *Providencia*, *Shigella*, and *Vibrio*, obtaining results ranging from strong to moderate antibacterial activity [89]. While Osorio et al. demonstrated that 6-PN (**1**) is active against Methicillin-resistant *Staphylococcus aureus bacteria* (MRSA). It exhibits a strong synergistic effect when combined with commonly used antibiotics such as vancomycin, ciprofloxacin, and methicillin, enhancing their effectiveness by 10 to 100 times [90] (Table 5). The finding that 6-PN (**1**) exhibits a strong synergistic effect with conventional antibiotics is highly significant. This synergy represents a clear, actionable pathway for future drug development. It is therefore crucial that future research prioritize the development of topical formulations of 6-PN (**1**) for localized infections, such as those caused by MRSA, where its dual action (direct antibacterial and antibiotic-enhancing) can be leveraged without the systemic bioavailability challenges.

#### 2.4.2. Antiviral Activity

Recurrent viral epidemics, particularly the recent COVID-19 pandemic, have driven scientists to search intensively for health-promoting supplements and drugs. Numerous studies have demonstrated the antiviral effects of polyphenols, particularly prenylflavonoids, on a variety of viruses. These include several RNA viruses, such as BVDV and HCV [116], as well as CMV and DNA viruses like HSV-1 and HSV-2 [117]. Additionally, phenolic compounds show antiviral activity against HIV-1 by inhibiting HIV-1 reverse transcriptase in vitro [118].

Bouback et al. investigated the effect of a crude extract from *H. lupulus* (hops) on the inhibition of Middle East respiratory syndrome coronavirus (MERS-CoV) and severe acute respiratory syndrome coronavirus 2 (SARS-CoV-2) replication in vitro, using Vero E6 cell lines (cells isolated from the kidney of an African green monkey). Hops extract showed very low toxicity on Vero E6 cells, with a CC_50_ of 23.25 µg/µL, while it demonstrated significant antiviral activity against MERS-CoV and SARS-CoV-2, with IC_50_ values of 0.18 µg/µL and 0.9 µg/µL, respectively. The crude extract achieved an inhibition rate of 84.6% for MERS-CoV and 80% for SARS-CoV-2. An in silico analysis revealed the presence of 6-PN (**1**) in the extract. This compound was found to inhibit the process of viral invasion into host cells by interfering with the viral spike protein’s ability to recognize the host cell receptor [119]. Herzog et al. reported that 6-PN (**1**), while not as active as xanthohumol (**4**), also targets SARS-CoV-2 PLpro, which is a promising therapeutic target because it contributes to both viral replication and the modulation of the immune system. This study found that 6-PN (**1**) exhibits marked antiviral activity against SARS-CoV-2. These findings support the possibility of developing new antiviral drugs [120]. Osorio et al. tested 6-PN (**1**) against SARS-CoV-2, obtaining antiviral activity IC_50_ = 7.3 μg/mL [90], while Morimoto et al. showed anti-Influenza activity of 6-PN (**1**) [53] (Table 5).

#### 2.4.3. Other Antimicrobial Activities

Additionally, 6-PN (**1**) has demonstrated antiparasitic activity against *Trypanosoma brucei* [115]. Human African trypanosomiasis, caused by this protozoan, is considered a neglected tropical disease that has a significant impact on human health, as it is fatal if left untreated [121]. Antifungal studies conducted on numerous filamentous fungi have shown rather low activity of 6-PN (**1**), with the exception of fungi of the genera *Microsporum, Mucor*, and *Trichophyton*, where growth inhibitory activity ranged from strong to moderate [10,90] (Table 5).

### 2.5. Impact on the Nervous System

Neurodegenerative diseases represent a growing burden on aging societies, making them a significant area of interest. Neurogenesis, the process by which new neurons are generated from neural stem cells in the adult brain, is partly regulated by sex hormones like estradiol. In animals, stimulation of neurogenesis by estradiol correlates with improved neurological function. Urmann et al. explored the effects of 8-PN (**2**), 6-PN (**1**), and related compounds on the in vitro differentiation of neuronal precursor cells. While prenylated flavanones **1** and **2** exhibit differing estrogenic activities, they both have the same effect on inducing differentiation in neural precursor cells [122].

GABA type A (GABA_A_) receptors for γ-aminobutyric acid are the primary inhibitory neurotransmitter receptors responsible for rapid inhibition in the basal part of the brain. These receptors belong to the ion channel family. They mediate rapid synaptic transmission and serve as important pharmacological targets for drugs, including anxiolytics and hypnotics [123]. In 1980, Hänsel et al. identified that 2-methyl-3-buten-2-ol, a degradation product of humulone and lupulone from hops, is responsible for the plant’s sedative and hypnotic properties by increasing the activity of γ-aminobutyric acid (GABA) [124]. Later, it was shown that 8-prenylnaringenin (**2**) also has a therapeutic effect, but this effect depends on the specific enantiomer (2*R* or 2*S*). The *2R*-8-PN (**2**) enantiomer shows a higher affinity (meaning a lower inhibition constant) for all tested transporters—serotonin, noradrenaline, and dopamine—than the 2*S*-8-PN (**2**) enantiomer [125]. Since 8-PN (**2**) naturally occurs in hops as the 2*S* enantiomer, these findings suggest that the synthesis of optically pure enantiomers of prenylated flavanones, such as 8-PN (**2**) or 6-PN (**1**), could be crucial for the pharmaceutical industry to develop more effective treatments.

Benkherouf et al. examined humulone interactions with other active hops compounds: 6-PN (**1**) and isoxanthohumol (IXN) on GABA-induced displacement of [^3^H]EBOB binding to native GABA_A_ receptors in rat brain membranes. They confirmed that humulone exhibits sedative/hypnotic effects and acts as a positive allosteric modulator of GABA_A_ receptors. In the presence of 3 mM GABA, 1 mM of humulone led to an additive potentiation of GABA-induced [^3^H]EBOB displacement in rat forebrain. The same results were obtained when testing 6-PN (**1**) and isoxanthohumol. Moreover, co-incubation of humulone with 6-PN (**1**) or isoxanthohumol, and with a combination of both 6-PN (**1**) and isoxanthohumol, significantly increased this potentiation [126]. These findings suggest that the neuroactivity of hops may result from the presence and interaction of other hop compounds, such as 6-PN (**1**) and/or isoxanthohumol, but not humulone alone. The results demonstrate a synergistic action of hop flavanones in stimulating the GABA_A_ receptor, emphasizing the role of 6-PN (**1**) and isoxanthohumol in enhancing the action of humulone on this receptor [126]. Other studies have shown that among the prenylflavonoids found in hops, 6-prenylnaringenin (**1**) is the most active positive allosteric modulator of GABA_A_ receptors. In comparison to xanthohumol (**4**), isoxanthohumol, and 8-prenylnaringenin (**2**), 6-PN (**1**) was found to be 8, 3, and 2 times more active, respectively [127,128].

An open-field rotary rod study in mice demonstrated that 6-PN (**1**) can cross the blood–brain barrier (BBB) without affecting locomotor activity. Literature data suggest that both 2*R* and 2*S* enantiomers of 6-PN (**1**), along with their derivatives, act as blockers of T-type calcium channels (T channels) Cav3.2. The blocking of these T channels has been shown to alleviate visceral and neuropathic pain without causing cardiovascular or behavioral side effects [129]. 6-PN (**1**) also shows promise for pain treatment [130,131].

The superior potency of 6-PN (**1**) as a GABA_A_ receptor positive allosteric modulator, being 2 to 8 times more active than its congeners, positions it as a prime therapeutic lead for conditions requiring CNS modulation (anxiety, insomnia, pain). Furthermore, its capacity to block T-type calcium channels (Cav3.2) to alleviate visceral and neuropathic pain provides a second, distinct mechanism for analgesic application. Future studies should focus on synthesizing optically pure enantiomers and derivatives to optimize both their GABA_A_ and T-channel activity, aiming for clinical trials to confirm their behavioral effects in vivo.

## 3. Synthesis

Although 6-prenylnaringenin (**1**) has attractive biological properties, its low concentration in plant extracts makes large-scale isolation economically impractical [132]. The various synthetic approaches developed have often suffered from low efficiency and multiple steps. While some methods have been modified to improve yields, the complex three-ring structure of flavonoids allows for prenylation at different positions, which complicates synthesis.

**Figure 3:**
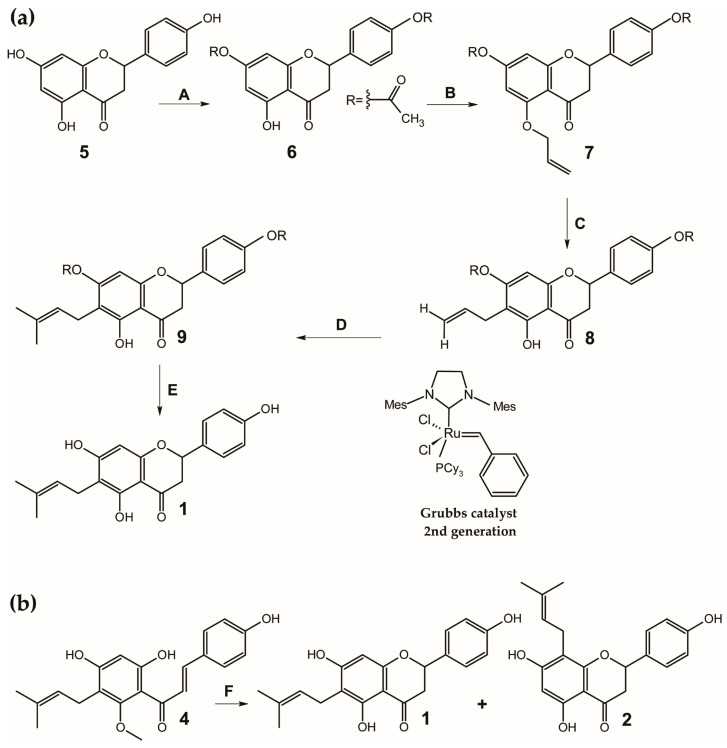
Synthesis of 6-prenylnaringenin (**1**) via: (**a**) europium(III)-catalyzed Claisen rearrangement and cross-metathesis [139] and (**b**) microwave irradiation [140]. Reagents, conditions and yields: (**A**) Ac_2_O 2 equivs, pyridine, room temperature, 89%; (**B**) allyl alcohol, PPh_3_, diethyl azodicarboxylate, THF, 0 °C to room temperature, 73%; (**C**) 10 mol% Eu(fod)_3_, CHCl_3_, 70 °C, 16.5 h, 74%; (**D**) isobutylene, 1 mol% Grubbs catalyst 2nd generation, benzene, room temperature 2 days, 78%; (**E**) MeOH, K_2_CO_3_ 0.3 equivs., 40 °C, 2 h, 88%; (**F**) lithium chloride, dimethylforamide, microwave irradiation, 198 °C, 9 min, 38%. **1**—6-prenylnaringenin (4′,5,7-trihydroxy-6-prenylflavanone), **2**—8-prenylnaringenin (4′,5,7-trihydroxy-8-prenylflavanone), **4**—xanthohumol (2′,4′,4-trihydroxy-6′-methoxy-5′-prenylchalcone), **5**—naringenin (4′,5,7-trihydroxyflavanone), **6**—4′,7,-diacetylnaringenin (4′,7-diacetoxy-5-hydroxyflavanone), **7**—4′,7-diacetyl-5-O-allylnaringenin (4′,7-diacetoxy-5-allyloxyflavanone), **8**—4′,7-diacetyl-6-allylnaringenin (4′,7-diacetoxy-6-allyl-5-hydroxyflavanone), **9**—7,4′-diacetyl-6-prenylnaringenin (4′,7-diacetoxy-5-hydroxy-6-prenylflavanone). Total yields: **5**→**1** 29%; **4**→**1** 38%.

6-PN (**1**) was first synthesized in 1978 [133,134,135]. However, the descriptions of the NMR spectra from that time contain discrepancies that have raised doubts about the reported structure [10]. One early method involved the condensation of naringenin with 2-methyl-but-3-en-2-ol in the presence of boron trifluoride etherate, which produced a mixture of three main fractions, one of which contained 6-PN (**1**) [135]. The total synthesis of 6-PN (**1**) has also been reported using an acetophenone derivative as a key intermediate [136]. A later pathway, reported by Tischer and Metz in 2006, aimed for complete regioselectivity at the C-6 position using a europium(III)-catalyzed Claisen rearrangement [137] followed by cross-metathesis [138,139], using relatively cheap and readily available naringenin (4′,5,7-trihydroxyflavanone) (**5**) as substrate for synthesis (Figure 3a). Despite these efforts, the yields of these early synthetic methods were not satisfactory. More recently, Urmann and Riepl developed a more efficient method using the design of experiment (DOE) and microwave synthesis. They achieved a high total yield of 76% by demethylating xanthohumol (**4**) with lithium chloride in dimethylformamide, which produced both 8-prenylnaringenin (**2**) (yield 38%) and 6-prenylnaringenin (**1**) (yield 38%) (Figure 3b) [140].

Another promising approach is through genetic engineering. Researchers successfully transferred the prenyltransferase gene (ShFPT) from *Streptomyces* sp. NT11 into *Escherichia coli*. The enzyme showed high selectivity for prenylating naringenin at the C-6 position. Under optimal conditions (pH 6.0 and 55 °C), this bioconversion method achieved a peak yield of 69.9 mg/L and an average yield of 4.0 mg/L/h after 16 h of incubation, demonstrating an effective biotechnological way to produce 6-PN (**1**) [141].

## 4. Conclusions

This review establishes 6-PN (**1**) as a multifaceted, high-priority therapeutic candidate distinguished by its potent neuroactive, synergistic antimicrobial, and unique chemopreventive profile.

6-PN (**1**) demonstrates a range of antimicrobial activities, including antibacterial, antiviral (e.g., against SARS-CoV-2), and antiparasitic effects. 6-PN (**1**) could serve as a core scaffold for developing new antimicrobials, especially through structural modifications such as halogenation of ring B. Research on the simultaneous administration of 6-PN (**1**) and antibiotics is also worth pursuing. Combining them could potentially reduce the required drug dosage, lower the risk of systemic toxicity, and increase treatment effectiveness, particularly when applied topically to avoid toxicity. Given the promising results from therapies using lupulone when antibiotic therapy fails, the antibacterial properties of 6-PN (**1**) are of significant interest.

6-PN (**1**) exhibits cytotoxic and antiproliferative effects on various cancer cell lines. It is considered a candidate for chemoprevention of non-hormone-dependent tumors and as a model structure for new therapies. However, to determine the usefulness of 6-PN (**1**) for cancer treatment, several key areas need further investigation. It is necessary to understand its potential toxic side effects on healthy tissues, detail its molecular mechanisms, and, most importantly, confirm its effects in vivo. Current laboratory data are insufficient to provide clinicians with clear guidelines for using 6-PN (**1**). While early short-term studies suggest that phytoestrogens might have a stimulatory effect in women at high risk for or with breast cancer, long-term studies are crucial to confirm their effects and to avoid potential side effects related to their weak estrogenic activity in patients with hormone-dependent cancers. Crucially, researchers need to verify whether 6-PN (**1**) acts as a chemopreventive agent or if it can produce a synergistic effect when combined with conventional therapies. Such an effect could potentially shorten the duration of therapy, limit side effects, and help prevent further cancer mutations.

The most promising avenue for 6-prenylnaringenin (**1**) lies in its capacity as a positive allosteric modulator of GABA_A_ receptors and its synergistic effect with traditional antibiotics. To translate these findings into clinical reality, research efforts must be directed toward the chemical modification of the 6-PN (**1**) core—leveraging modern synthetic techniques to improve the water solubility and oral bioavailability that currently limit its therapeutic potential. Furthermore, targeted in vivo studies are urgently needed to confirm its behavioral effects (anxiolytic, sedative, analgesic) and establish its long-term safety profile, particularly concerning its anti-cancer chemopreventive role and non-proliferative effects on ER-positive tumors. Concurrently, the accelerated development of topical or localized and targeted delivery systems should be pursued to capitalize on the powerful antibiotic synergy demonstrated against drug-resistant pathogens like MRSA. Given the amount of ongoing research and modern technological capabilities, the future for 6-prenylnaringenin (**1**) looks promising, provided that research shifts focus from mere characterization to targeted development based on its distinct non-hormonal therapeutic advantages.

## Figures and Tables

**Table 1:** The content of 6-Prenylnaringenin (**1**) in different plant materials.

Source	Content in Dry Mass [%]
*Sophora* sp.	detected [9,17,18]
*Wyethia* sp.	0.051 [19]
*Lupinus luteus*	detected [13]
*Glycyrrhiza glabra*	0.48 [14]
*Psoralea corylifolia*	0.068 [20]
*Humulus lupulus*	0.004 [16]
Hop extract	0.170 [7]

**Table 2:** Content of 6-prenylnaringenin (**1**) and total content of prenylflavonoids in various types of beers and herb tea [32,38].

Type of Beverages	6-PN (1) (C.V., n = 2) [μg/L]	6-PN (1) [μg/L] (±SD)	Total Prenylflavonoids [μg/L]
Lager/pilsner	31–38 (1.0–4.0)	14.2 (0.58)	460–750 [32]/693 [38]
American Porter	560 (2.2)		2900 [32]
Dunkel (dark beer)		355 (3.1)	2375 [38]
American Hefeweizen	11 (2.1)		330 [32]
Strong ale	200 (0.3)		4000 [32]
India Pale Ale (IPA)	146 (2.4)	368 (15.9)	2446 [38]
Double IPA		421 (9.7)	2331 [38]
Imported stout	170 (0.7)		2680 [32]
Coffe stout		351 (11.2)	2445 [38]
Helles (pale lager)		46.7 (0.4)	1260 [38]
Imported lager	1 (28)	14.2 (0.1)	40 [32]/693 [38]
Imported pilsner	22–55 (2.0–7.7)	22.8 (0.2)	680 [32]/1051 [38]
Bock		156 (3.0)	1879 [38]
Doppelbock beer A		203 (2.4)	1346 [38]
Doppelbock beer B		63.6 (0.6)	1614 [38]
Wheat bock beer		91.2 (3.2)	771 [38]
Festbeer (festival beer)		51.8 (0.90)	1234 [38]
Hopped beer A		278 (5.6)	2042 [38]
Hopped beer B		120 (6.7)	1993 [38]
IPA alcohol free		104 (3.2)	1332 [38]
Non-alcohol beer	7 (8.2)		120 [32]
Herb tea	4 (5.4)	14.2 (0.58)	20 [32]

SD—standard deviation, C.V.—Coefficient of variation = 100 × standard deviation/mean concentration.

**Table 3:** Natural health products and dietary supplements containing 6-PN (**1**) for managing menopausal symptoms or as an alternative to hormone replacement therapy [8].

Product	Recommended Dose ^1^	Country of Manufacture
Nature’s Own™ MenoSleep	2 tablets	Australia
AOR™ Advanced Series Estro Detox™	2 capsules	Canada
Life Extension^®^ Natural Female Support	1 capsule	United States
BioCeuticals^®^ MenoPlus 8-PN™	1 tablet	Australia
Garden of Life^®^ Oceans 3™ Healthy Hormones^®^	3 softgels	United States

^1^ The content of 6-PN (**1**) is given on the label of natural health products and dietary supplements.

**Table 4:** In vitro activity of 6-prenylnaringenin (**1**) as a potential anticancer agent.

Biological Activity	Cell Line/Enzyme	Concentration: IC_50_^A^ (μM) or CC_50_^B^ (μg/mL)	Reference
Antiproliferative Activity	PC-3	18.4 **^A^** ± 1.2	[86]
	DU 145	29.1 **^A^** ± 1.1	
	T-47D	16.01 **^A^** ± 3.74	[87]
	MCF7	43.25 **^A^** ± 4.37	
	MDA-MB-231	62.64 **^A^** ± 19.54	
	A2780	44.16 **^A^** ± 14.71	
	A2780cis	81.73 **^A^** ± 17.68	
	HT-29	64.61 **^A^** ± 17.07	
	UO.31, PC-3	-	[88]
Inhibition of EGFR, MEK, ERK kinase	H4IIE	33 ± 5 μM	[20]
	Hct116	34 ± 4 μM	
	C6	>50 μM	
Cytotoxic activity	HSC-2	22 **^B^** ± 0.065	[89]
	HSG	32 **^B^** ± 0.094	
	HGF	35 **^B^** ± 0.103	
	MDA-MB-231	53.94 **^B^** ± 9.66	[90]
	B16-F10	49.14 **^B^** ± 3.38	
	MEF	48.45 **^B^** ± 3.44	
Inhibition of cellular histone deacetylases (HDACs)	SK-MEL-28	100 µM	[91]
	BLM	100 µM	
Induced caspase-independent form of cell death	DU 145	>200 µM	[92]
	PC-3	<200 µM	
Induction of quinone reductase (QR) activity	Hepa-1c1c7	-	[93]
Inhibition of metabolic activation of procarcinogens	P450 CYP1A	0.09 **^A^**	[94]
	P450 CYP1A1	0.63 ± 0.08	
	P450 CYP1B1	0.21 ± 0.02	

**A** = IC_50_ (μM)—the concentration of 6-PN (**1**) which inhibits 50% of the tested cells population or halfmaximal inhibitory concentration for enzyme activity; **B** = CC_50_—cytotoxic concentration in µg./mL required to reduce the number of viable cells by 50%; PC-3, DU 145—human prostate cancer cell lines; T-47D, MCF7, MDA-MB-231—human breast cancer cell lines; A2780—human ovarian cancer cell line; A2780cis—human cisplatin-resistant ovarian cancer cell line; HT-29—colon cancer cell line; UO.31—renal carcinoma cell line; SK-MEL-28—human melanoma cell line; BLM—human metastatic melanoma cell line; Hepa-1c1c7—mouse hepatoma cell line; CYP—cytochrome P450; HSC-2, HSG—human oral tumor cell lines; HGF—human gingival fibroblast; B16-F10—mouse melanoma cell line; MEF—primary mouse embryonic fibroblast; H4IIE—rat hepatocellular carcinoma cell line; Hct116—human colon carcinoma cell line; C6—rat C6 glioma cell line; (-) no numerical data available in the literature.

**Table 5:** Antimicrobial, antiviral, antifungal, and antiparasitic activities of 6-prenylnaringenin (**1**).

Biological Activity	Organism/Virus	Concentration: MIC_50_^A^, CC_50_^B^, EC_50_^C^, IC_50_^D^ [μg/mL]	Reference
Anti-G-positive bacteria activity	*Bacillus subtilis* VB1	>100 **^A^**	[89]
	*Staphylococcus aureus* 8531	50 **^A^**	
	*Staphylococcus aureus* 8530	50 **^A^**	
	*Staphylococcus typhimurium* 4	>10 **^A^**	
	*Staphylococcus typhimurium* 57	>100 **^A^**	
	*Staphylococcus typhimurium* 59	>100 **^A^**	
	*Staphylococcus aureus* 6571	25 **^A^**	
	*Staphylococcus aureus* MRSA 97-7	5 **^A^**	[90]
	*Staphylococcus aureus* MRSA 622-4	25 **^A^**	
	*Staphylococcus aureus* ATCC 6538	10 **^A^**	
Anti-G-negative bacteria activity	*Klebsiella* spp. 14	>100 **^A^**	[89]
	*Providencia* spp. 1	>100 **^A^**	
	*Shigella dysenteriae* 1	50 **^A^**	
	*Shigella sonnei* 2	>100 **^A^**	
	*Vibrio cholerae* 865	25 **^A^**	
	*Escherichia coli* R832	25 **^A^**	
	*Escherichia coli* ROW	>100 **^A^**	
	*Helicobacter pylori*	>100 **^A^**	
	*Escherichia coli*	>200 **^A^**	[10]
Antifungal activity	*Aspergillus flavus* ATCC9170	>250 **^A^**	[90]
	*Aspergillus fumigatus* ATCC26934	>250 **^A^**	
	*Aspergillus niger* ATCC9029	>250 **^A^**	
	*Candida albicans* ATCC10231	>250 **^A^**	
	*Candida albicans*	>200 **^A^**	[10]
	*Cryptococcus neoformans* ATCC32264	125 **^A^**	[90]
	*Cryptococcus neoformans* clinical isolates	250 **^A^**	
	*Microsporum gypseum* CCC115	62.5 **^A^**	
	*Trichophyton mentagrophytes* ATCC9972	62.5 **^A^**	
	*Trichophyton rubrum* CCC113	62.5 **^A^**	
	*Trichophyton mentagrophytes*	3.13 **^A^**	[10]
	*Trichophyton rubrum*	3.13 **^A^**	
	*Fusarium oxysporum*	>200 **^A^**	
	*Mucor rouxianous*	50 **^A^**	
Antiviral activity	Anti-HIV activity	125 **^B^**	[89]
		>200 **^C^**	
		<1 (SI (CC_50_/EC_50_))	
	Anti-Influenza	38 ± 4.7 **^D^**	[53]
	SARS-CoV-2	7.3 **^D^**	[90]
Antiparasitic activity	*Trypanosoma brucei*	11.4 ± 0.34 **^A^**	[115]

**A** = MIC_50_—minimum concentration of 6-PN (**1**) which inhibits 50% of the tested cells population (μg/mL); **B** = CC_50_—cytotoxic concentration in µg/mL required to reduce the number of viable cells by 50%, **C** = EC_50_—inhibitory concentration in µg/mL required to inhibit the cytopathic effects by 50%; **D** = IC_50_—inhibitory concentration in µg/mL required to reduce viral replication by 50%. SI—selectivity index; MRSA—Methicillin-resistant *Staphylococcus aureus.*

## Data Availability

No new data were created or analyzed in this study. Data sharing is not applicable to this article.
